# Human Outbreak of Trichinellosis Caused by *Trichinella papuae* Nematodes, Central Kampong Thom Province, Cambodia

**DOI:** 10.3201/eid2608.191497

**Published:** 2020-08

**Authors:** Yannick Caron, Sotharith Bory, Michel Pluot, Mary Nheb, Sarin Chan, Sang Houn Prum, Sun Bun Hong Lim, Mala Sim, Yi Sengdoeurn, Ly Sovann, Virak Khieu, Isabelle Vallée, Hélène Yera

**Affiliations:** Institut Pasteur, Phnom Penh, Cambodia (Y. Caron, M. Sim);; Calmette Hospital, Phnom Penh (S. Bory, M. Pluot, M. Nheb, S. Chan);; Preah Ket Mealea Hospital, Phnom Penh (S. H. Prum);; Kampong Thom Province Hospital, Kampong Thom, Cambodia (S.B.H. Lim);; Ministry of Health, Phnom Penh (Y. Sengdoeun, L. Sovann, V. Khieu);; Agence Nationale Sécurité Sanitaire Alimentaire Nationale, Maisons-Alfort, France (I. Vallée);; Paris University, Paris, France (H. Yera)

**Keywords:** Trichinella papuae, trichinellosis, nematodes, parasites, outbreak, food safety, biopsy, serologic analysis, rDNA intergenic spacer region, Kampong Thom Province, Cambodia

## Abstract

In September 2017, a severe trichinellosis outbreak occurred in Cambodia after persons consumed raw wild pig meat; 33 persons were infected and 8 died. We collected and analyzed the medical records for 25 patients. Clinical signs and symptoms included myalgia, facial or peripheral edema, asthenia, and fever. We observed increased levels of creatine phosphokinase and aspartate aminotransferase­, as well as eosinophilia. Histopathologic examination of muscle biopsy specimens showed nonencapsulated *Trichinella* larvae. A *Trichinella* excretory/secretory antigen ELISA identified *Trichinella* IgM and IgG. Biopsy samples were digested and larvae were isolated and counted. PCR for the 5S rDNA intergenic spacer region and a multiplex PCR, followed by sequencing identified the parasite as *Trichinella papuae*. This species was identified in Papua New Guinea during 1999 and in several outbreaks in humans in Thailand. Thus, we identified *T. papuae* nematodes in humans in Cambodia.

Trichinellosis is a parasitic disease caused by nematodes of the genus *Trichinella* and acquired by ingestion of raw or undercooked meat from infected animals (nonuminant mammals, birds, and reptiles). Approximately 11 million persons worldwide might be infected by *Trichinella* spp. (*1*). Numerous animals species (≈100), including humans, can be infected and the most common source of human trichinellosis is meat from pig or wild pig (*Sus scrofa*) (*2*). Therefore, this zoonotic disease is not only a public health hazard but also represents an economic problem in porcine animal production and food safety. Some countries in Europe implemented a *Trichinella* monitoring program (*3*,*4*), and a *Trichinella*-free pig production pilot program has also been set up in the United States (*5*). Nevertheless, estimation of the effect of trichinellosis in developing countries with reference to public health and social and economic costs is difficult.

Recent studies on the genetic diversity, zoogeographic, and epidemiologic features within this genus resulted in a revised *Trichinella* taxonomy comprising 10 species (13 genotypes): encapsulated (*T. spiralis*, *T. nativa*, *T. britovi*, *T. murrelli*, *T. nelsoni*, *T. patagoniensis*, and *T. chanchalensis*) and nonencapsulated (*T. pseudospiralis*, *T. papuae*, and *T. zimbabwensis*) (*2*,*6*,*7*). In Southeast Asia, relatively few human outbreaks were recorded, but it has been calculated that >40 million persons are at risk for *Trichinella* infection in China (*8*). The presence of *Trichinella* antibodies in asymptomatic persons in a rural population of Cambodia (unknown location) was described (*9*), and another study reported 24 cases of *Trichinella* spp. in Khmer immigrants living in the United States (*10*). In Vietnam (*11*) and Laos (*12*), several outbreaks were reported involving *T. spiralis* nematodes. However, in Thailand, several human outbreaks involving *T. spiralis*, *T. pseudospiralis*, and *T. papuae* nematodes were reported (*13*,*14*).

The *T. papuae* nematode (genotype T10) is one of the most recently discovered species and was first detected in sylvatic swine of Papua New Guinea in 1999 by using molecular tools (*15*). In addition to being a nonencapsulated species, *T. papuae* nematodes are known to use mammals and reptiles (mainly crocodiles but also to a lesser extent caimans, turtles, and lizards) as hosts (*16*). Humans acquired the parasite by the consumption of raw meat from domestic animals, wild pigs, saltwater crocodiles, and turtles (*17*). This species was identified in Thailand in humans during outbreaks in 2006 (*14*) and 2007 (*13*) and in a patient returning from Malaysia in 2011 (*18*). The objective of this study was to report and describe a documented human outbreak of trichinellosis caused by *T. papuae* nematodes in Cambodia.

## Materials and Methods

### Study Area

At the end of September 2017, a trichinellosis outbreak occurred near Chak Tav village, Mean Rith commune, Sandan District in Kampong Thom Province, Cambodia. This location is situated in the Prey Lang Forest (13°07′N, 105°30′E), which is a nature reserve forest covering 3,600 km^2^.

### Data Sources and Ethics

During this outbreak, we collected medical and epidemiologic data from Kampong Thom Provincial Hospital and 2 hospitals (Preah Ket Mealea and Calmette) in Phnom Penh. The Pasteur Institute of Cambodia had an official agreement from the Communicable Disease Control Department of the Ministry of Health of Cambodia to study this outbreak, including collection of medical records of the patients, as well as remaining biologic samples. Because this retrospective study respected anonymity, did not involve any patient intervention, and was conducted in the frame of control and national epidemiologic surveillance, ethics agreement or informed consent was not necessary.

### Serodiagnosis

Serodiagnosis was performed for most (11/12) patients hospitalized in Preah Ket Mealea Hospital by a laboratory in Vietnam (https://www.medic-lab.com). This laboratory used an excretory/secretory antigen–based ELISA for detection of IgM (negative result if optical density <1.0) and IgG (negative result if optical density <0.3).

### Histopathologic Analysis

For all 13 patients at Calmette Hospital, we obtained 2 biopsy specimens from the deltoid and biceps or gastrocnemius muscles, preserved them in 10% phosphate-buffered formalin, and sent them to the Pathology Department of the hospital. Formalin-fixed samples were dissected, embedded in paraffin (at 56°C), cut with a rotary microtome preset for a thickness of 4 μm, and stained with hematoxylin phloxine saffron. Histopathologic preparations were then microscopically examined.

### Detection of *Trichinella* larvae

Sections from slides, as well as remaining paraffin blocks for each of the sample were sent to the National Reference Laboratory for Human Trichinellosis at Cochin Hospital (Paris, France). Paraffin was eliminated from tissues after incubation in Histo-Clear (National Diagnostics, https://www.nationaldiagnostics.com) and then washed in ethanol and phosphate-buffered saline. We digested a total of 60–115 mg of muscle tissue by using pepsin/HCl solution for 30 min–1 hr at 45°C (*4*). Larvae were then collected, counted, and identified on the basis of morphologic characteristics (*19*).

### Molecular Identification of Larvae

Molecular identification of larvae was also performed in the National Reference Laboratory for Human Trichinellosis. DNA was extracted from 30 mg of the deparaffinated samples by using the tissue QIA Amp DNA Mini Kit (QIAGEN, https://www.qiagen.com) and from larvae by using the NucliSens easyMAG Kit (bioMérieux, https://www.biomerieux.com) according to manufacturer’s instruction. Pretreatment with proteinase K (>600 mAU/mL) was performed overnight at 56°C with shaking at 1,200 vibrations/min, and DNA was eluted in 100 μL of distilled water. DNA was also extracted from a *T. nativa* larval suspension and used as a positive control. A total of 200 μL of sterile water was used as a negative control.

### Conventional PCR for *Trichinella* rDNA Intergenic Spacer Region

Primers and the protocol used to amplify the 5S rDNA intergenic spacer region of *Trichinella* were described (*20*). In brief, we used 0.5 μmol/L of each primer in a 50-μL reaction containing 1.5 mmol/L MgCl_2_, 200 μM of each dNTP, 1.5 U of AmpliTaq Gold (Applied Biosystems, https://www.thermofisher.com) and 5 μL of extracted DNA. Reactions were run in a Perkin-Elmer Thermocycler (https://www.perkinelmer.com) under the following conditions: 95°C for 5 min for 1 cycle; 95°C for 30 s, 55°C for 30 s and 72°C for 40 s for 42 cycles; followed by a 15-min final extension step at 72°C. The PCR products were separated by 1% agarose gel electrophoresis at 90 mA and stained with ethidium bromide.

### Multiplex PCR for *Trichinella* spp.

We used 5 primer pairs in a multiplex PCR as described (*21*,*22*): primer set I, expansion segment V (ESV) target locus; primer set II, internal transcribed spacer region 1 (ITS1) target locus; primer set III, ITS1 target locus; primer set IV, ITS2 target locus; and primer set V, ITS2 target locus. Reactions were performed in 25 μL of 2XGOTaq HotStartGreen MasterMix (Promega, https://www.promega.com), 6.3 μL of nuclease-free water, 1.7 μL of total primers, and 17 μL of extracted DNA. We performed the PCR cycle as follows: a predenaturation and polymerase activation step at 95°C for 2 min, then 35 amplification cycles (denaturation at 95°C for 10 s, hybridization at 55°C for 30 s, and elongation at 72°C for 30 s), and a final elongation step at 72°C for 5 min. PCR products were isolated separated by using 2% agarose gel electrophoresis.

We sequenced PCR products by using appropriate primers (Eurofins-MWG, https://www.eurofins.fr) and aligned them by using BioEdit version 7.0.9 (https://www.biodeit.software.informer.com). We also performed a BLASTn (https://blast.ncbi.nlm.nih.gov) search. Sequences obtained in this study were deposited in GenBank, and accession numbers are listed in the Results.

## Results

### Course of the Outbreak

After consumption of raw meat from a wild pig during July–August 2017, a total of 33 persons (rangers, workers from the Ministry of Environment, and villagers) became infected by the nematode. The number and precise date of meal(s) were unknown. Eight persons died according to Khmer official sources (*23*) and others (*24*). On August 1, the first case-patient was hospitalized at Kampong Thom Provincial Hospital because of severe clinical signs and symptoms of trichinellosis (abdominal pain; fever; myalgia; swelling of the face, arms, and legs; joint pain; headache; and pruritus).

The Ministry of Health immediately sent a Rapid Response Team of the Department of Communicable Disease Control to the area and conducted a retrospective epidemiologic survey (results are not available) and a risk assessment (resulting in a moderate level of risk) (*23*). Nine patients were admitted to Kampong Thom Provincial Hospital, and 8 patients were transferred to Calmette Hospital. Seventeen other patients were admitted directly to Calmette Hospital (5) or Preah Ket Mealea Hospital (12).

Little information was available for the 8 persons who died: 6 had refused to be transferred to Phnom Penh and were given mainly traditional medicine, 1 died during transport from Kampong Thom to Phnom Penh, and 1 died in Calmette hospital. Several information sessions were held in Kampong Thom Province to warn the population and provide advice on sanitation. Furthermore, a workshop was also organized that involved national authorities and the World Health Organization in December 2017. Complete medical records of the 25 patients hospitalized in Phnom Penh hospitals were collected and analyzed.

### Epidemiologic, Laboratory, and Clinical Features

We provide epidemiologic, laboratory, and clinical features for the 25 available case-patients (Table). The first patient hospitalized in Kampong Thom Provincial Hospital arrived on August 1, and most (25/33) patients were referred to Phnom Penh during September 6–October 17. The average age of the patients was 34.5 years (range 20–50 years), and the male:female ratio was 24:1.

**Table:** Characteristics for 25 patients during a human outbreak of trichinellosis caused by *Trichinella papuae* nematodes, Cambodia*

Pt no.	Age, y/sex	Clinical signs or symptoms	Length of hospital stay, d	Leu†	Eos†	Neu†	CPK†	LDH†	AST†	Biopsy result	IgM/IgG	PCR/PCR/Seq‡
1	26/M	Mu, As, Oe, Dy	12	23	3	21	1,210	433	97	+	ND	–/+/ND
2	31/M	Mu, Fe, Hd, Oe, Dy	9	23	4	18	1,216	577	310	+	ND	–/+/ND
3	20/M	Mu, Fe, Cg, Dy	19	28	2	23	784	1,890	89	+	ND	+/+/+
4	34/M	Mu, As, Oe, Dy	14	21	2	19	1,499	469	59	+	ND	+/+/+
5	25/M	Mu, As	4	9	2	4	809	589	74	+	ND	+/+/+
6	26/M	Mu, Fe, Oe	14	20	2	19	340	584	51	+	ND	+/+/+
7	35/M	Mu, Hd, Gi, As, Oe	10	29	3	22	1,265	2,300	104	+	ND	−/−/ND
8	43/M	Mu, Fe, As, Oe	12	15	3	12	1,007	1,940	70	+	ND	–/+/ND
9	50/M	Mu, Oe	7	13	1	10	1,263	830	87	+	ND	–/+/ND
10	27/M	Mu, As, Oe	13	13	3	10	862	592	86	+	ND	–/+/ND
11	35//M	Mu, As, Oe	13	23	3	19	1,367	2,030	115	+	ND	-/+/ND
12	40/M	Mu, Fe, As, Oe, Cg, Dy	15	18	2	16	1,091	536	127	+	ND	–/+/ND
13	28/F	Mu, Fe, Gi, As, Oe, Dy, Cg	18	36	2	35	827	2,540	117	+	ND	–/+/+
14	46/M	Mu, Fe, Gi	17	22	3	20	1,521	248	100	ND	–/+	ND
15	22/M	Mu, Fe, Hd, As, Gi, Cg	8	16	1	11	110	137	30	ND	−/−	ND
16	40/M	Mu, Fe, Gi, As, Oe	18	18	10	12	1340	342	73	ND	ND	ND
17	42/M	Mu, Fe, Gi, As	16	25	5	20	260	162	44	ND	–/+	ND
18	36/M	Mu, Fe, Hd, As, Dy	16	17	2	12	899	ND	281	ND	–/+	ND
19	42/M	Mu, Fe, As	16	24	2	17	796	ND	161	ND	+/+	ND
20	26/M	Mu, Fe, As	16	21	8	17	340	ND	70	ND	+/+	ND
21	26/M	Mu, Hd, As Oe	25	18	1	16	1,073	291	94	ND	+/+	ND
22	34/M	Mu, Fe, As, Dy	25	21	4	20	622	303	89	ND	+/+	ND
23	41/M	Mu, Fe, Hd, Gi, As	15	16	1	13	866	246	170	ND	+/+	ND
24	42/M	Mu, Fe, Hd, Gi, As, Dy	15	18	1	15	524	ND	113	ND	–/+	ND
25	45/M	Mu, Fe, Hd, Gi	7	11	2	15	320	ND	154	ND	–/+	ND

*As, asthenia; AST, aspartate aminotransferase (reference range <37 U/L); Cg, cough; CPK, creatine phosphokinase (reference range 15–130 U/L); Dy, dyspnea; Eos, eosinophils (reference range 0.2–0.7 × 10^9^ cells/L); Fe, fever; Gi, abdominal pain, diarrhea, and nausea; Hd, headache; Leu, leukocytes (reference range 4–9 × 10^9^ cells/L); LDH, lactate dehydrogenase (reference range 225–450 U/L); Mu, muscle pain and swelling; ND, not determined; Neu, neutrophils; Oe, facial or periorbital edema; Pt, patient; Seq, sequencing; –, negative; +, positive. †Values are the highest readings obtained during hospitalization. ‡Positive conventional PCR for *Trichinella* rDNA intergenic spacer region/multiplex PCR for *Trichinella* spp./sequencing.

According to the case definition criteria (*17*), a total of 23 cases could be classified as confirmed because of myalgia (or fever, edema, or diarrhea), eosinophilia (or increased level of muscle enzymes), positive serologic results, or muscle biopsy reports. Patient 15 was classified as having a suspected case because only myalgia, fever, and diarrhea were observed. Patient 16 was classified as having a highly probable case because he had myalgia, fever, edema, diarrhea, eosinophilia, and increased levels of muscle enzymes (creatine phosphokinase [CPK] and aspartate aminotransferase [AST]).

Every patient hospitalized in Phnom Penh reported muscle pain and swelling (25/25; 100%), followed by asthenia (18/25; 72%); fever (17/25; 68%), facial or periorbital edema (13/25; 52%); abdominal pain, diarrhea, and nausea (9/25; 36%); headache (8/25; 32%); dyspnea (7/25; 28%); and cough (3/25; 12%). All 25 patients had a history of consumption of wild pig meat. A total of 96% (24/25) of patients had leukocytosis (range 4–23 × 10^9^ cells/L) and eosinophilia (range 1–10 × 10^9^ cells/L). Patient 5 had no leukocytosis but did have eosinophilia.

Enzyme levels indicative of muscle damage were also increased in 96% (24/25) of the patients: CPK (range 110–1,499 U/L) and AST (range 30–310 U/L). CRP (range 6.3–310.3 mg/L) was increased for 68% (17/25) of the patients. Furthermore, levels of lactate dehydrogenase (range 137–2,300 U/L) was also increased for 62% (16/25) of the patients. All 13 biopsies performed in Calmette Hospital were positive for *Trichinella* larvae, and 91% (10/11) of serologic test results for patients at Preah Ket Mealea Hospital were positive for *Trichinella* IgG and IgM. In both hospitals in Phnom Penh, patients were hospitalized for an average for 14 days (range 4–26 days). Hepatosplenomegaly was observed during abdominal ultrasound examination for 47% (9/19) of the patients. Chest radiography, electrocardiography, and echocardiography for all patients showed typical results. Patient 2 died from multiorgan failure (probably caused by septicemia), despite intensive care.

### Identification and Confirmation of Larvae

**Figure 1:**
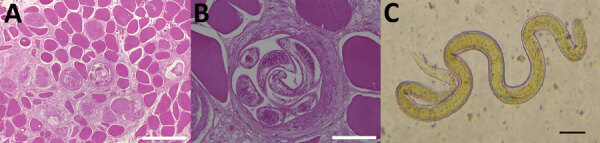
*Trichinella* larvae samples from patients in Cambodia. A) Transverse section of a muscle (bicep) biopsy specimen from patient 13 showing a nonencapsulated *T. papuae* stage 1 nematode larva in the center of the specimen (hematoxylin phloxine saffron stain; scale bar = 200 μm). B) Higher magnification view of the same biopsy specimen showing the coelomyarian muscle structure and stichosome of *Trichinella* larvae (scale bar = 50 μm). C) *T. papuae* stage 1 nematode larva after deparaffinization and artificial digestion of muscle biopsy specimen (scale bar = 50 μm).

Transverse sections of nonencapsulated larvae morphologically consistent with *Trichinella* sp. stage L1 larvae were identified by histopathologic analysis (Figure 1, panels A and B). A diffuse infiltration of larvae was visible in all slides, accompanied by abnormally clear cytoplasm, vacuolization, and disorganization of myofibrils or total necrosis of striated muscle. The interstitial connective tissue was edematous and had few inflammatory cells. There were no encysted larvae with a hyaline wall of a cyst or organized fibrosis. After digestion of the remaining biopsy sample with pepsin/HCl, *Trichinella* sp. larvae were found in 92% (12/13) of the patients. The mean ± SD number of larva in the 60-mg tissue sample was 17 ± 13 (minimum 4 and maximum 41). We also observed a nonencapsulated larvae of a *Trichinella* sp. after the pepsin/HCl digestion (Figure 1, panel C).

**Figure 2:**
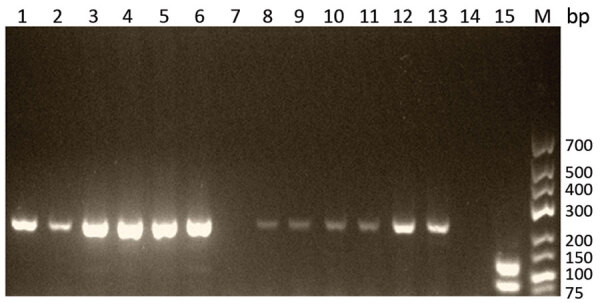
Gel electrophoresis (2% agarose) of products of *Trichinella* multiplex PCR using samples from patients in Cambodia. Lanes 1–6, patients 1–6: samples were extracted from muscle tissue and show the 240-bp band typical for *Trichinella papuae* nematodes. Lane 7, patient 7: sample was extracted from muscle tissue and showed no band. Lanes 8–13, patients 8–13: samples were extracted from larvae and show the 240-bp band typical for *T. papuae* nematodes. Lane 14, negative control; lane 15, positive control (*T. nativa* showing the expected 127-bp band); lane M, molecular mass ladder.

The 5S rDNA intergenic spacer region of *Trichinella* was amplified from 4/13 muscle biopsy specimens (for patients 3, 4, 5, and 6; GenBank accession numbers MN158145–8). The sequences alignment showed 10 positions of single-nucleotide polymorphisms on 794 nt between the isolates. The BLASTn matched with the highest percentage of identity (range 97.8%–98.0%) and the lowest E value (0.0) with the sequence of *T. papuae* nematodes (GenBank accession no. AY845861.1). Similarly, the multiplex PCR showed (Figure 2) a typical pattern for *T. papuae* nematodes (240-bp band) for 12/13 samples. The amplified ESV gene was sequenced (for patients 4, 6, and 13; GenBank accession nos. MN158365–7). Alignment showed 1 nt change over 238 nt between the isolates. The BLASTn matched with the highest percentage of identity (range 98.3%–100%) and the lowest E value (range 4.10 × 10^−114^ to 1.10 × 10^−118^) for *T. papuae* nematodes from crocodiles in Papua New Guinea (GenBank accession nos. FJ493493.1 and FJ493494.1).

The ESV gene could not be amplified in 1 sample (patient 7). In addition, no larvae were observed in this sample after digestion with pepsin/HCl.

### Treatment and Clinical Follow-up

All patients were given albendazole (800 mg or 15 mg/kg/d for a minimum of 10 days) and glucocorticosteroid (prednisolone) (60 mg or 1 mg/kg/d for 5 days). Supportive treatments such as painkillers for myalgia or intravenous fluid and electrolytes against hypovolemia and metabolic disorders were also administered. To our knowledge, 24 patients recovered, but it was not possible to obtain additional information from them.

## Discussion

We report an outbreak of *T. papuae* nematode infection in Cambodia and provide epidemiologic, molecular, laboratory, and clinical data. The most probable mode of infection was consumption of wild pig meat, although the possibility of some infections being caused by consumption of reptile meat (e.g., Siamese crocodiles [*Crocodylus siamensis*] and lizards) present in the Prey Lang Forest, cannot be completely rule out (*14*). Several human outbreaks of trichinellosis (caused by nonencapsulated *Trichinella* species) through consumption of raw soft-shelled turtle (*Pelodiscus sinensis*) meat in South Korea (*25*) and Taiwan (*26*) have been described. *T. papuae* nematodes might have been the causative species in these infections because it is often found in reptile meat.

The central location of Kampong Thom Province in Cambodia (far from regions in Thailand that had previously described outbreaks) and the fact that a wild pig was the source of infection could indicate that Kampong Thom Province is a region to which *T. papuae* nematodes are endemic. Comparing the only 2 ESV sequences deposited in GenBank from *T. papuae* nematodes in Papua New Guinea (*27*) and those from this study showed an identity of 100%. Comparing of the only 5S rDNA intergenic spacer region sequence from *T. papuae* nematodes (GenBank accession no. AY845861.1) and those from this study showed an identity of ≈98%. A higher intraspecies variation was observed between the *T. papuae* sequences from Cambodia by using 5S rDNA intergenic spacer region analysis.

It is likely that outbreaks characterized by a low number of ingested larvae, few patients, or mild clinical signs and symptoms are not correctly diagnosed and consequently, public health services are not informed. This situation was also described in Papua New Guinea (*28*), Laos (*11*), and Vietnam (*12*). Therefore, trichinellosis is probably underdiagnosed, and the high number of deaths in this outbreak alerted authorities. Because no previous trichinellosis cases were recorded, we can hypothesize that sporadic cases are the rule.

The average yearly incidence of trichinellosis in humans worldwide is probably ≈10,000 cases, and the mortality rate is ≈0.2% (*29*). Complications can be life-threatening and affect mainly the elderly. Their frequency is variable and dependent on the epidemics; it can sometimes involve <30% of cases with neurologic complications and 5%–20% for cardiac and vascular complications. During this outbreak, the mortality rate was high (24%, 8/33). In comparison, the observed mortality rate during the epidemic in France during 1985 was 5 deaths/1,000 persons (*30*). However, *T. spiralis* nematodes, not *T. papuae*, nematodes, were presumed to be the causative agent. Furthermore, to our knowledge, deaths caused by *T. papuae* nematodes have not been reported.

The quantity of ingested larvae (*2*), the quantity of alcohol (*31*,*32*) consumed during a meal, the nature of the host, and the species of *Trichinella* (*33*) are some of the parameters linked to virulence. However, the quantity of meat consumed (positively correlated with virulence) and the quantity of alcohol consumed (negatively correlated with virulence) was not known for our case-patients. Furthermore, little information was available for the patients who died. Infection with *Trichinella* species was not medically proven for 7 of the officially recorded fatal cases and this factor confounded accurate interpretation of the epidemiology of this outbreak. A high number of ingested larvae, a late diagnosis in the first hospital, or the choice of some patients to use traditional medicine instead of modern medicine could be a reason for this unusual high mortality rate. Moreover, access to medical care in Cambodia is limited and it was recently pointed out that the national healthcare system at the same time enables, encourages, and sanctions unregulated medical practice (*34*). In the case of fatal trichinellosis, cardiac or neural complications occurring in the first 2 weeks of infection are most common (*19*). Complications can be avoided by the early combined use of albendazole and prednisolone (*35*). These drugs were given to the 25 documented case-patients, even to the patient who eventually died.

Cultural factors, such as consumption of traditional dishes that contain raw or undercooked meat, play a major role in the transmission of *Trichinella* nematodes to humans. Because patients were referred to hospitals in Phnom Penh for a long period (78 days) after the first case, we can hypothesize that several contaminated meals could have been taken from the same meat source prepared in different ways (sun-dried meat is commonly eaten in Cambodia). However, samples from the meals were not available for examination. In Cambodia, common popular beliefs associated a strength transfer for man eating raw wild pig meat, but this type of food is widely rejected by woman because of its pronounced taste (M. Sim, Institut Pasteur, Phnom Penh, Cambodia, pers. comm., 2017 Dec 1) and can probably explain the observed unbalanced sex ratio. Other parasites, such as *Sarcocystis* spp. (*36*) or *Taenia* spp. (*37*), which are also transmitted by consumption of raw meat, are also endemic in Cambodia.

On the basis of clinical signs and symptoms, all patients arrived in Phnom Penh during the acute phase of trichinellosis because 100% had myalgia and swelling, 72% had asthenia, and 68% had fever. Furthermore 96% had moderate eosinophilia, and increased levels of muscle enzymes (AST, CPK, and lactate dehydrogenase) (*19*). Patients 15 and 16 could not be classified as having confirmed trichinellosis because of the absence of biopsy and serologic results. Nevertheless, albendazole-based treatment cured the patients. None of these patients had any complications. We hypothesize that most deaths occurred because of complications. The medical record of patient 2 showed that he underwent surgery (appendectomy) 2 weeks before his death (on September 21). It is likely that the course of the *Trichinella* infection and the surgery played a major role in fatal multiorgan failure. Moreover, the appendicitis symptoms could also have been related to trichinellosis.

ELISA is the recommended diagnostic technique for trichinellosis and is best used in combination with confirmatory immunoblotting (Western blot) (*19*). In this outbreak, ELISA was only performed for 44% (11/25) of the patients. Only 1 of these patients had a negative serologic result. Delayed seroconversion until 60 days postinfection has been described (*38*). The result of a second serology test would have been useful, but it was not possible to obtain a new blood sample from the patient. IgG and IgM can persist up to 1 year and occasionally even longer (11 years and 19 years have been reported) (*39*). Although a specific excretory/secretory antigen ELISA was used, we cannot eliminate the possibility of false-positive results caused by cross-reactions with other endemic parasites (i.e., *Toxocara* spp., *Strongyloides stercoralis*, or *Toxoplasma* spp.) (*40*). Nevertheless, when considered in the context of consumption of raw wild pig meat, clinical symptoms and positive results for biopsies and molecular biology assays leave little doubt regarding the accuracy of serologic results.

Further investigations are needed to answer the questions raised by this outbreak and to identify the life cycle of *T. papuae* nematodes in Cambodia. It could be useful to determine the serologic status of inhabitants in the area of the outbreak and the prevalence of *Trichinella* infection in wild pigs and reptiles found in the nearby forest. The sylvatic and domestic life cycles of the parasite are probably linked, and it could be useful to investigate the spectrum of domestic hosts to avoid further outbreaks.

In Cambodia, a *Trichinella* species is now accurately identified as causing an outbreak. This finding should encourage authorities and involved sectors to implement measures to limit the effect of this major zoonotic parasite and its spreading to (domestic) pigs herds. Consumption of raw meat (particularly wild pig) must be avoided. Furthermore, this outbreak highlights that the *T. papuae* nematode should be reevaluated as a potential virulent species for humans.

## References

[1] Dupouy-Camet J. Trichinellosis: a worldwide zoonosis. Vet Parasitol. 2000;93:191–200. 10.1016/S0304-4017(00)00341-1 11099837

[2] Gottstein B, Pozio E, Nöckler K. Epidemiology, diagnosis, treatment, and control of trichinellosis. Clin Microbiol Rev. 2009;22:127–45. 10.1128/CMR.00026-08 19136437 PMC2620635

[3] European Food Safety Authority. The community summary report on trends and sources of zoonoses, zoonotic agents, antimicrobial resistance and foodborne outbreaks in the European Union in 2006. European Food Safety Authority Journal. 2006;94:167–74.

[4] The European Commission. Commission Implementing Regulation (EU) 2015/1375 of 10 August 2015 laying down specific rules on official controls for *Trichinella* in meat (text with EEA relevance); 2015 [cited 2019 May 10]. https://eur-lex.europa.eu/legal-content/EN/TXT/?uri=CELEX%3A32015R1375

[5] Pyburn DG, Gamble HR, Wagstrom EA, Anderson LA, Miller LE. Trichinae certification in the United States pork industry. Vet Parasitol. 2005;132:179–83. 10.1016/j.vetpar.2005.05.051 15993000

[6] Krivokapich SJ, Pozio E, Gatti GM, Prous CLG, Ribicich M, Marucci G, et al. *Trichinella patagoniensis* n. sp. (Nematoda), a new encapsulated species infecting carnivorous mammals in South America. Int J Parasitol. 2012;42:903–10. 10.1016/j.ijpara.2012.07.009 22921601

[7] Sharma R, Thompson PC, Hoberg EP, Brad Scandrett W, Konecsni K, Harms NJ, et al. Hiding in plain sight: discovery and phylogeography of a cryptic species of Trichinella (Nematoda: Trichinellidae) in wolverine (*Gulo gulo*). Int J Parasitol. 2020;50:277–87. 10.1016/j.ijpara.2020.01.003 32171846

[8] Bai X, Hu X, Liu X, Tang B, Liu M. Current research of trichinellosis in China. Front Microbiol. 2017;8:1472. 10.3389/fmicb.2017.01472 28824597 PMC5539376

[9] Pozio E. Taxonomy of *Trichinella* and the epidemiology of infection in the Southeast Asia and Australian regions. Southeast Asian J Trop Med Public Health. 2001;32(Suppl 2):129–32. 12041576

[10] Stehr-Green JK, Schantz PM. Trichinosis in Southeast Asian refugees in the United States. Am J Public Health. 1986;76:1238–9. 10.2105/AJPH.76.10.1238 3752328 PMC1646684

[11] Van D N, Thi Nga V, Dorny P, Vu Thrung N, Ngoc Minh P, Trung Dung BD, et al. Trichinellosis in Vietnam. Am J Trop Med Hyg. 2015;92:1265–7. 10.4269/ajtmh.14-0570 PMC4458836 25846295

[12] Barennes H, Sayasone S, Odermatt P, De Bruyne A, Hongsakhone S, Newton PN, et al. A major trichinellosis outbreak suggesting a high endemicity of *Trichinella* infection in northern Laos. Am J Trop Med Hyg. 2008;78:40–4. 10.4269/ajtmh.2008.78.40 18187783 PMC7611085

[13] Kusolsuk T, Kamonrattanakun S, Wesanonthawech A, Dekumyoy P, Thaenkham U, Yoonuan T, et al. The second outbreak of trichinellosis caused by *Trichinella papuae* in Thailand. Trans R Soc Trop Med Hyg. 2010;104:433–7. 10.1016/j.trstmh.2009.12.005 20427064

[14] Khumjui C, Choomkasien P, Dekumyoy P, Kusolsuk T, Kongkaew W, Chalamaat M, et al. Outbreak of trichinellosis caused by *Trichinella papuae*, Thailand, 2006. Emerg Infect Dis. 2008;14:1913–5. 10.3201/eid1412.080800 19046519 PMC2634638

[15] Pozio E, Owen IL, La Rosa G, Sacchi L, Rossi P, Corona S. *Trichinella papuae* n.sp. (Nematoda), a new non-encapsulated species from domestic and sylvatic swine of Papua New Guinea. Int J Parasitol. 1999;29:1825–39. 10.1016/S0020-7519(99)00135-6 10616929

[16] Pozio E. The broad spectrum of *Trichinella* hosts: from cold- to warm-blooded animals. Vet Parasitol. 2005;132:3–11. 10.1016/j.vetpar.2005.05.024 15970384

[17] Pozio E, Zarlenga DS. New pieces of the *Trichinella* puzzle. Int J Parasitol. 2013;43:983–97. 10.1016/j.ijpara.2013.05.010 23816802

[18] Intapan PM, Chotmongkol V, Tantrawatpan C, Sanpool O, Morakote N, Maleewong W. Molecular identification of *Trichinella papuae* from a Thai patient with imported trichinellosis. Am J Trop Med Hyg. 2011;84:994–7. 10.4269/ajtmh.2011.10-0675 21633039 PMC3110362

[19] Dupouy-Camet J, Murrell KD. FAO/WHO/OIE Guidelines for the surveillance, management, prevention and control of trichinellosis. Dupouy-Camet J, Murrell KD, editors. Paris: Food and Agriculture Organization of the United Nations, World Health Organization, World Organisation for Animal Health; 2007 [cited 2020 May 20]. http://www.trichinellosis.org/uploads/FAO-WHO-OIE_Guidelines.pdf

[20] De Bruyne A, Yera H, Le Guerhier F, Boireau P, Dupouy-Camet J. Simple species identification of *Trichinella* isolates by amplification and sequencing of the 5S ribosomal DNA intergenic spacer region. Vet Parasitol. 2005;132:57–61. 10.1016/j.vetpar.2005.05.026 15992998

[21] Zarlenga DS, Chute MB, Martin A, Kapel CM. A multiplex PCR for unequivocal differentiation of all encapsulated and non-encapsulated genotypes of *Trichinella.* Int J Parasitol. 1999;29:1859–67. 10.1016/S0020-7519(99)00107-1 10616932

[22] Pozio E, La Rosa G. PCR-derived methods for the identification of *Trichinella* parasites from animal and human samples. Methods Mol Biol. 2003;216:299–309. 12512373 10.1385/1-59259-344-5:299

[23] Ministry of Health. Cambodia. Cases of trichinellosis in Kampong Thom Province; 2017 [cited 2020 May 20]. http://www.cdcmoh.gov.kh/298-2017-09-26-02-39-19

[24] Bo X. Cambodia confirms outbreak of trichinellosis in central Kampong Thom Province. Xinhua; September 26, 2017 [cited 2020 May 20]. http://www.xinhuanet.com/english/2017-09/26/c_136639855.htm

[25] Jeong JT, Seo M, Hong S-T, Kim YK. An outbreak of trichinellosis by consumption of raw soft-shelled turtle meat in Korea. Korean J Parasitol. 2015;53:219–22. 10.3347/kjp.2015.53.2.219 25925182 PMC4416374

[26] Lo YC, Hung CC, Lai CS, Wu Z, Nagano I, Maeda T, et al. Human trichinosis after consumption of soft-shelled turtles, Taiwan. Emerg Infect Dis. 2009;15:2056–8. 10.3201/eid1512.090619 19961701 PMC3044530

[27] Pozio E, Owen IL, Marucci G, La Rosa G. Inappropriate feeding practice favors the transmission of *Trichinella papuae* from wild pigs to saltwater crocodiles in Papua New Guinea. Vet Parasitol. 2005;127:245–51. 10.1016/j.vetpar.2004.09.029 15710525

[28] Owen IL, Gomez Morales MA, Pezzotti P, Pozio E. *Trichinella* infection in a hunting population of Papua New Guinea suggests an ancient relationship between *Trichinella* and human beings. Trans R Soc Trop Med Hyg. 2005;99:618–24. 10.1016/j.trstmh.2005.03.005 15922379

[29] Pozio E. World distribution of *Trichinella* spp. infections in animals and humans. Vet Parasitol. 2007;149:3–21. 10.1016/j.vetpar.2007.07.002 17689195

[30] Dupouy-Camet J, Talabani H, Ancelle T. [Trichinellosis] [in French]. Rev Prat. 2010;60:159–64. 20225547

[31] Steven WM, Kumar SN, Stewart GL, Seelig LL Jr. The effects of ethanol consumption on the expression of immunity to *Trichinella spiralis* in rats. Alcohol Clin Exp Res. 1990;14:87–91. 10.1111/j.1530-0277.1990.tb00452.x 2178479

[32] Na HR, Zhu X, Stewart GL, Seelig LL Jr. Ethanol consumption suppresses cell-mediated inflammatory responses and increases T-helper type 2 cytokine secretion in *Trichinella spiralis*-infected rats. Alcohol Clin Exp Res. 1997;21:1179–85. 10.1111/j.1530-0277.1997.tb04435.x 9347076

[33] Sadaow L, Intapan PM, Boonmars T, Morakote N, Maleewong W. Susceptibility of laboratory rodents to *Trichinella papuae.* Korean J Parasitol. 2013;51:629–32. 10.3347/kjp.2013.51.6.629 24516265 PMC3916449

[34] Gryseels C, Kuijpers LM, Jacobs J, Grietens KP. When “substandard” is the standard, who decides what is appropriate? Exploring healthcare provision in Cambodia. Crit Public Health. 2019;29:460–72. 10.1080/09581596.2019.1591614

[35] Pozio E, Gomez Morales MA, Dupouy-Camet J. Clinical aspects, diagnosis and treatment of trichinellosis. Expert Rev Anti Infect Ther. 2003;1:471–82. 10.1586/14787210.1.3.471 15482143

[36] Khieu V, Marti H, Chhay S, Char MC, Muth S, Odermatt P. First report of human intestinal sarcocystosis in Cambodia. Parasitol Int. 2017;66:560–2. 10.1016/j.parint.2017.04.010 28476340

[37] Adenuga A, Mateus A, Ty C, Borin K, Holl D, San S, et al. Seroprevalence and awareness of porcine cysticercosis across different pig production systems in south-central Cambodia. Parasite Epidemiol Control. 2017;3:1–12. 10.1016/j.parepi.2017.10.003 29774294 PMC5952675

[38] Owen IL. Parasitic zoonoses in Papua New Guinea. J Helminthol. 2005;79:1–14. 10.1079/JOH2004266 15831107

[39] Harms G, Binz P, Feldmeier H, Zwingenberger K, Schleehauf D, Dewes W, et al. Trichinosis: a prospective controlled study of patients ten years after acute infection. Clin Infect Dis. 1993;17:637–43. 10.1093/clinids/17.4.637 8268344

[40] Gómez-Morales MA, Ludovisi A, Amati M, Cherchi S, Pezzotti P, Pozio E. Validation of an enzyme-linked immunosorbent assay for diagnosis of human trichinellosis. Clin Vaccine Immunol. 2008;15:1723–9. 10.1128/CVI.00257-08 18827188 PMC2583519

